# Supplementary material for: The effect of mountaineering on the grit of college students: an empirical study
Source: PeerJ. 2025 Mar 12;13:e19086. doi: 10.7717/peerj.19086 (PMC11910154; doi:10.7717/peerj.19086)
Supplement: Supplemental Information 2 [file peerj-13-19086-s002.docx]

Evidence that permission is not required

We extensively read literature related to Grit-O scale and Lake Louise scale before conducting our research, there is no permission requirement to use this scale in all literature. Moreover, in the following two original papers, the authors or organizations hope or welcome future researchers to directly use these two scales to advance research in the field.

[1] Duckworth, A. L., Peterson, C., Matthews, M. D., & Kelly, D. R. (2007). Grit: perseverance and passion for long-term goals. Journal of personality and social psychology, 92(6), 1087–1101. <https://doi.org/10.1037/0022-3514.92.6.1087>

[2] Roach, R. C., Hackett, P. H., Oelz, O., Bärtsch, P., Luks, A. M., MacInnis, M. J., Baillie, J. K., & Lake Louise AMS Score Consensus Committee (2018). The 2018 Lake Louise Acute Mountain Sickness Score. High altitude medicine & biology, 19(1), 4–6. <https://doi.org/10.1089/ham.2017.0164>
